# Absence of Cytochrome P450-1b1 Increases Susceptibility of Pressure-Induced Axonopathy in the Murine Retinal Projection

**DOI:** 10.3389/fcell.2021.636321

**Published:** 2021-03-05

**Authors:** Naseem Amirmokhtari, Brian D. Foresi, Shiv S. Dewan, Rachida A. Bouhenni, Matthew A. Smith

**Affiliations:** ^1^Department of Pharmaceutical Sciences, Northeast Ohio Medical University, Rootstown, OH, United States; ^2^Integrated Pharmaceutical Medicine Graduate Program, Northeast Ohio Medical University, Rootstown, OH, United States; ^3^Rebecca D. Considine Research Institute, Vision Center, Akron Children’s Hospital, Akron, OH, United States

**Keywords:** glaucoma, retinal ganglion cell, microbead occlusion model, nodes of Ranvier, axonal transport disruption

## Abstract

Mutations in the cytochrome P450-1B1 (Cyp1b1) gene is a common genetic predisposition associated with various human glaucomas, most prominently in primary congenital glaucoma (PCG). The role of Cyp1b1 in the eye is largely unknown, however, its absence appears to drive the maldevelopment of anterior eye structures responsible for aqueous fluid drainage in murine models. Nevertheless, vision loss in glaucoma ultimately results from the structural and functional loss of retinal ganglion cells (RGCs). Cyp1b1’s influence in the development and support of retinal ganglion cell structure and function under normal conditions or during stress, such as elevated ocular pressure; the most common risk factor in glaucoma, remains grossly unknown. Thus, to determine the role of Cyp1b1 in normal retinal projection development we first assessed the strucutrual integrity of RGCs in the retina, optic nerve, and superior colliculus in un-manipulated (naïve) Cyp1b1-knockout (Cyp1b1^–/–^) mice. In addition, in a separate cohort of Cyp1b1^–/–^ and wildtype mice, we elevated and maintained intraocular pressure (IOP) at glaucomatous levels for 5-weeks, after which we compared RGC density, node of Ranvier morphology, and axonal transport between the genotypes. Our results demonstrate that naïve Cyp1b1^–/–^ mice develop an anatomically intact retinal projection absent of overt glaucomatous pathology. Following pressure elevation, Cyp1b1^–/–^ accelerated degradation of axonal transport from the retina to the superior colliculus and altered morphology of the nodes of Ranvier and adjacent paranodes in the optic nerves. Together this data suggests the absence Cyp1b1 expression alone is insufficient to drive murine glaucomatous pathology, however, may increase the vulnerability of retinal axons to disease relevant elevations in IOP.

## Introduction

Glaucoma is a group of heterogeneous neuro-ophthalmologic conditions that impair vision through the functional disruption and eventual degeneration of retinal ganglion cells (RGCs), the neuronal substrates responsible for eye-brain communication (Davis et al., 2016; Quigley and Broman, 2006). Glaucoma is most often attributed to the aging adult (Davis et al., 2016; Tham et al., 2014), however, befalls pediatric and adolescent populations (Kaur et al., 2011), thereby placing it in a unique group of neurodegenerative conditions that afflict populations on both ends of the lifespan.

Primary congenital glaucoma (PCG) is the most prevalent form of pediatric glaucoma manifesting at birth or within 3 years of age (Aponte et al., 2010). PCG is characterized most often by elevated intraocular pressure (IOP), buphthalmos (enlarged globe), and significant maldeveloped ocular drainage structures (i.e., trabecular meshwork and iridocorneal angle) (Hoskins et al., 1984; Libby et al., 2003). Much like other forms of glaucoma, the etiology of PCG remains unknown but likely arises from an orchestration of genetic and post-translational factors. PCG can be difficult to diagnose and if not managed effectively will result in progressive vision loss which can have critical ramifications on a child’s overall development and long-term quality of life (Mandal et al., 2004; Khitri et al., 2012).

Genetic mapping of PCG populations over the last decade has identified recessive inheritance of several mutations in the cytochrome P450-1B1 (Cyp1b1) gene on the GLC3A locus (Sarfarazi et al., 1995; Akarsu et al., 1996) as a common predisposition. This has led many to assert Cyp1b1 as a causative gene for PCG. Interestingly, additional evidence suggests the involvement of Cyp1b1 mutations in several other forms of glaucoma including juvenile and adult primary open angle glaucoma (POAG) (Vincent et al., 2002; Su et al., 2012). Cyp1b1 is a membrane bound protein located in the endoplasmic reticulum or the inner mitochondrial membrane of cells found in most parts of the body (Stoilov et al., 2001; Bansal et al., 2014). Its general somatic involvement is in both endogenous and exogenous metabolism of xenobiotics and steroid synthesis (Murray et al., 2001). The role of Cyp1b1 in the eye is not well understood, however, its mRNA and protein expression in various ocular tissues including the cornea, ciliary body, trabecular meshwork, and the retina is evident (Muskhelishvili et al., 2001; Bejjani et al., 2002; Doshi et al., 2006).

The majority of studies seeking to understand the role of Cyp1b1 in the eye and its influence on the onset of PCG have focused primarily on signaling pathways integral in anterior eye structure development necessary for aqueous humor drainage. Cyp1b1 is involved in the metabolism of vitamin A (retinol) to the bioactive retinoic acid (RA). RA serves as a signaling molecule during a number of developmental and physiological processes, playing multiple roles during embryonic ocular and retinal development (Molotkov et al., 2006; Matt et al., 2008; Vasiliou and Gonzalez, 2008). Of the few murine studies conducted, deletion of Cyp1b1 *in vivo* yielded modest dysgenic anterior ocular drainage structures (trabecular meshwork and ciliary body) resembling defects seen in human PCG patients (Libby et al., 2003; Teixeira et al., 2015). While understanding the role of Cyp1b1 in anterior eye structure maldevelopment and causative signaling mechanisms holds significant value in understanding the onset and development of PCG; an avenue that remains overlooked is the contribution of Cyp1b1 in the retina and retinal projection dysfunction and degeneration underlying glaucomatous pathophysiology.

Glaucoma including PCG, involves an increased sensitivity of RGCs to changes in IOP. This increased sensitivity to elevations in IOP alters the axons of the RGCs (axonopathy) which encompass but are not limited to cytoskeletal alterations (Wilson et al., 2016), synaptic hypertrophy (Smith et al., 2016), aberrant morphology of axonal nodes of Ranvier (Smith et al., 2018) and defects in axonal transport (Dengler-Crish et al., 2014). The latter two appearing as the earliest pathological manifestations preceding overt degeneration of RGC axons in the optic nerve and somal loss in the retina (Smith et al., 2016). These pre-degenerative axonopathies represent key factors in the pathophysiological sequalae of glaucoma, which likely drive or follow changes in RGC physiology that is equally necessary for maintaining proper eye-brain communication (Baltan et al., 2010; Risner et al., 2018; Smith et al., 2018). To our knowledge no prior examination has been completed to determine the integrity of the retinal projections in the absence of Cyp1b1 under normal and stressed conditions (i.e., pathological elevation in IOP).

To address this gap in understanding of the role of Cyp1b1 in glaucoma, we compared the progression of RGC axonopathy in Cyp1b1^–/–^ and wildtype mice following pathological elevation in IOP using the magnetic microbead occlusion model. We found more pronounced RGC axonal transport deficits and abnormal node of Ranvier morphometry in Cyp1b1^–/–^ compared to wildtypes subjects following 5-weeks IOP elevation. We propose that the absence of Cyp1b1 expression alone is insufficient to drive murine glaucomatous pathology, however, the lack of its expression may increase the vulnerability of retinal neurons to disease relevant mechanisms following pathological elevations in IOP.

## Materials and Methods

### Subjects

Adult mixed sex Cyp1b1^–/–^ [129S6.129 × 1(B6)-Cyp1b1^TM1*Gonz*^/Mmnc] mice were obtained from the Mutant Mouse Resource and Research Center (MMRRC) at JAX (Jackson Laboratories) and genotyped before experimentation to confirm the transgene. These mice have a targeted mutation caused by a disrupted coding sequence associated with exon three of the Cyp1b1 gene. Age-matched 129S6.129 × 1(B6) mice were used as wildtype controls (Buters et al., 1999; Libby et al., 2003). Mice were maintained in the Comparative Medicine Unit at Northeast Ohio Medical University on a 12-hour light/dark cycle with standard rodent chow available *ad libitum*. All experimental procedures were approved by the Northeast Ohio Medical University Institutional Animal Care and Use Committee and conducted in accordance with the Guide for Care and Use of Laboratory Animals published by the National Institutes of Health.

### Groups, Sample Size, and Ages

Thirty-six mice were used for this study to create equivalent groups of mice based on genotype, and experimental conditions. For initial studies assessing Cyp1b1^–/–^ optic projection development a total combined cohort of twelve Cyp1b1^–/–^ (*n* = 6) and wildtype (*n* = 6) subjects were used. Each retina, optic nerve, and corresponding contralateral superior colliculus (SC) were analyzed as independent measures within each animal, as glaucomatous pathophysiology is known to differentially affect each eye and projection (Schlamp et al., 2006; Crish et al., 2010). Therefore, from the total cohort of twelve subjects, a total of 24 (*n* = 12 per group) optic projections were collected and analyzed. All tissues were collected in 4–6-month old subjects across both genotypes.

For subsequent studies aimed at determining the impact of Cyp1b1^–/–^ in the optic projection under conditions of ocular stress (i.e., induced ocular hypertension), an additional cohort of twenty-four 8–10 month old Cyp1b1^–/–^ (*n* = 12) and wildtype (*n* = 12) subjects were utilized. Each retina, optic nerve, and corresponding contralateral superior colliculus were analyzed as separate, independent measures within each animal. The left eye/projections across both genotype groups underwent microbeads occlusion to induced ocular pressure elevation constituting the “hypertensive” subgroup while the right eye/projection remained un-elevated, “normotensive” to serve as internal sham control.

### Intraocular Pressure Elevation Induction in Subjects

Intraocular pressure was raised to glaucomatous levels in our Cyp1b1^–/–^ and wildtype subjects using the microbead occlusion method as described in Lambert et al. (2019) and Smith et al. (2018) (Figure 2). Briefly, twelve Cyp1b1^–/–^ mice and twelve wildtype subjects were anesthetized using inhaled isoflurane (3% to induce and 1.5% to maintain sedation) and secured in a custom-made mount to reduce head and body movement during procedure. Baseline IOP readings were taken using a tonometer (TonoLab, Icare). Topical tropicamide (1%) was applied to both eyes for pupillary dilation, and a glass pipette (borosilicate glass capillaries 1.0/0.75 mm OD/ID pulled to 150-μm diameter; World Precision Instruments) attached to a Micro4 MicroSyringe Pump Injection system (World Precision Instruments) was used to deliver 1 μl (2.4 × 106 beads/ml) of COMPEL magnetic microspheres (mean diameter 7.90 μm; Bangs Laboratories Inc.) into the anterior chamber of the left eye for each animal. After completion of injections, a small neodymium magnet (Amazing Magnets) was used to pull beads to the margin of the eye. Each animal received a sham injection in the right eye with sterile saline used in place of microspheres as a procedural control. Animals were allowed to recover and IOP readings were recorded at 24- and 72-h and each week post injection for a 5-week survival time. After 5-weeks of IOP elevation, subjects underwent anterograde transport labeling procedures as detailed in section “Anterograde Axonal Transport Labeling” before being sacrificed following procedures detailed in section “Tissue Collection and Preparation.”

### Anterograde Axonal Transport Labeling

In order to assess the integrity of RGC anterograde axonal transport mechanisms in our subjects, we used axonal transport labeling techniques as we previously described (Dengler-Crish et al., 2014; Smith et al., 2016). Anterograde transport labeling was performed in all subjects across genotype and conditions. Subjects were placed prone in a stereotaxic device (Stoelting, Wood Dale, IL, United States) equipped with a nose cone that delivered 2.5% isoflurane at 0.8 ml/min. Injections (1.5 μl) of 0.1% cholera toxin subunit B (CTB) conjugated to Alexa Fluor-488 (Molecular Probes, C-34775) in sterile phosphate buffered saline (PBS) were administered into the vitreal chamber of each eye using a 33-ga needle attached to a 25 μl Hamilton syringe.

### Tissue Collection and Preparation

All subjects were sacrificed via an overdose of Beuthanasia-D (300 mg/kg, i.p.) and transcardially perfused with PBS followed by 4% paraformaldehyde (PFA). Retinas, optic nerves, and brains were dissected and submerged into 4% PFA for an additional post-fixation step. Post-fixation time was dependent on the tissue type. Retinas underwent 30-min post-fixation, whereas optic nerves and brains were post-fixed overnight. After post-fixation, tissues were cryoprotected in 20% sucrose/PBS overnight. Using a freezing sliding microtome, 50 μm-thick coronal serial sections through the rostral-caudal extent of the superior colliculus, and 15 μm-thick longitudinal optic nerve sections were collected. Retina were dissected from the eye and prepared as flattened whole-mounts.

### Examining the Structural Integrity of the Retinal Projections

In order to assess whether any anatomical differences exist regarding RGCs in the retinal-brain projections of the Cyp1b1^–/–^ mouse, we used immunohistochemistry to label all components of the RGCs including somas in the retina, nodes of Ranvier in the optic nerve, and the distal projection axons and terminals in the superior colliculus. Retina, optic nerve, and brain tissues were incubated at 37°C for 1 h in a blocking solution containing 5% normal donkey serum and 0.1% Triton-X 100 in PBS, followed by overnight incubation at room temperature in a primary antibody solution containing 3% serum, 0.1% Triton. Primary antibodies used were: Brn3a (mouse 1:500; Santa Cruz Biotenchonology) to label the retinal ganglion cell nuclei, Caspr (mouse 1:500; Millipore) for axon paranode junctions, Nav1.6 (rabbit 1:500; Alamone) for axonal sodium channels at the nodes of Ranvier, ERRβ (rabbit 1:500; Sigma-Aldrich) for terminating retinal ganglion cell axons, Vglut2 (guinea pig 1:500; Synaptic Systems) for the synaptic terminals in the superior colliculus. After primary antibody incubations, tissues were washed, and then incubated at room temperature for 2 h in CF-Dye conjugated (488, 594, and 647 dyes) secondary antibody solution (Biotium) at a dilution of 1:200 in 1% serum/0.1% Triton/PBS. Following the final washes, tissues were mounted onto slides and coverslipped with ProLong Glass mounting media (Thermo Fisher).

### Microscopy

All images were collected on a Zeiss Axio Imager M2 equipped with a digital high-resolution camera (Hamamatsu Flash4.0 V3 Digital CMOS; Japan), motorized Z and X–Y stage and an Apotome. Two structured illumination systems using 20×/0.8, 63×/1.4, and 150×/1.35 NA Plan-Apochromat objectives (Zeiss, Jena, Germany); all ran from the Zen operating software equipped with deconvolution and extended-depth-of-field modules. For retina imagining, multi-frame z-stacked acquisition was used to create a montage image composite of the entire retinal surface (Simons et al., 2021). For the optic nerve, optical sections (0.3 μm) through longitudinal sections were collected using the Apotome 2. Images were collected at two distinct regions at the center length of each nerve (Smith et al., 2018). For the superior colliculus, every third section of the serial right superior colliculus section was imaged.

### Image Analysis

All analysis detailed in the section was conducted by multiple participants that were blinded to the genotype and condition from which the tissue was derived as slides were de-identified and assignment of an arbitrary numerical sequence was used to allow for minimal identification across subjects and study groups.

To determine the density of the RGC somas in the retina of Cyp1b1^–/–^ compared to wildtype controls we used ImagePro (Media Cybernetics; Rockville, MD, United States) software to count and calculate Brn3a-labeled RGCs in the retina. Densities (cells/mm^2^) were derived by dividing the total number of Brn3a-positive RGCs counted in each retina by the total area of that retina.

To assess the integrity of axonal transport, and intactness of distal RGC axons in naïve, normotensive and hypertensive Cyp1b1^–/–^ mice, we quantified CTB, ERRβ, and Vglut2 signal density in multiple slices of the superior colliculus using a custom-written macro for NIH ImageJ. We set background intensity for each superior colliculus section by selecting a region of non-retinorecipient (i.e., periaqueductal gray) for comparison with the retinorecipient superior colliculus. We selected only the retinorecipient layers of the superior colliculus and binned pixels running from medial to lateral superior colliculus. The number of pixels within bins with CTB, ERRβ, or Vglut2 signal brighter than background was divided by the total number of pixels in the bin to provide a signal density for each serial section. The signal density from each serial superior colliculus section was averaged for each animal and compared across all genotypes and manipulation.

Node and paranode lengths, were derived from raw image z-stacks analyzed with the Zen 2 software analysis module as previously described (Smith et al., 2018). Node of Ranvier length was defined as the minimum distance separating adjacent Caspr terminal ends with the paranode length defined as the distance across a single unilateral Caspr signal determined from z-stacks of nodes that were linearly oriented to the imaging plane.

### Statistical Analysis

Statistical data analysis was performed using IBM SPSS 26 Software. Raw data were screened for outliers, normalcy, and homogeneity of variance. We used two-way factorial analyses of variance (ANOVA) to determine differences in the magnitude of transport and structural label in the superior colliculus and retina between genotypes. Only CTB label was analyzed across subjects between genotypes in microbead occlusion experiments. Average node and paranode lengths were compared between genotype/microbead occlusions groups using factorial ANOVA models with Bonferroni’s corrected pairwise comparisons to elucidate subgroup differences. Microbead occlusions IOP data were analyzed using an omnibus mixed model within subjects (ANOVA) to determine whether IOP changed in each eye after model induction as a function of bead implantation (hypertensive) or control saline injection (normotensive).

## Results

### CYP1B1^–/–^ Develop Structurally Intact Visual Projections

**FIGURE 1:**
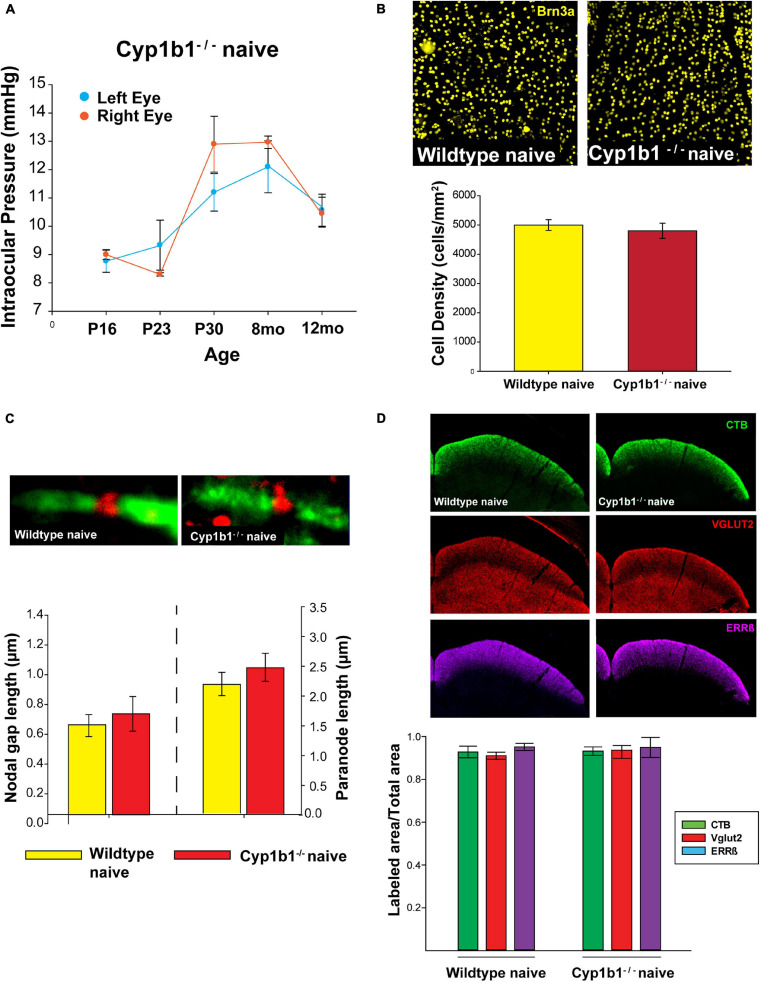
Cyp1b1^–/–^ develop structurally intact visual projections. **(A)** Cyp1b1^–/–^ ocular pressure readings across the lifespan (P16 to 12-mo, *n* = 6) maintained within normal physiological range in both eyes. **(B)** Retinal ganglion cell density in naïve wildtype vs. Cyp1b1^–/–^ mice. High magnification retinal whole-mount images immunostained for Brn3a, a marker for RGC nuclei. Naïve Cyp1b1^–/–^ (red; *n* = 12) RGC densities do not differ from naïve wildtype retina (yellow; *n* = 12). **(C)** Cyp1b1^–/–^ nodes of Ranvier appear absent of major morphometric changes in the node (Nav1.6, red; *n* = 200) and paranode (Caspr, green; *n* = 200). **(D)** A 50 μm coronal cross section through the superior colliculus of a Cyp1b1^–/–^ mouse that received an intravitreal injection of cholera toxin-B conjugated –alexafluor 488 (CTB488, green) and immunostained for VGlut2 (red, RGC synapses) and estrogen related receptor-β (magenta, RGC axon + axon terminals). Graph depicts superior colliculus label density (labeled area/total area) for each marker. No differences in label density were present between Cyp1b1^–/–^ and wildtype (*n* = 24).

As summarized in Figure 1, we did not find any major differences in the structural integrity of RGC at the level of the retina, optic nerve, or superior colliculus in naïve Cyp1b1^–/–^ mice compared to wildtype subjects. Using tonometry to record IOPs in both left and right eyes in Cyp1b1^–/–^ mice aging from postnatal day 16 (P16) to 12-months there was no significant difference in IOP between the left and right eyes across all ages (Figure 1A; *F*_1_,_28_ = 0.18, *p* > 0.1). Qualitatively, recorded IOPs from Cyp1b1^–/–^ mice at all examined ages did not extend outside of physiological ranges of 10–15 mmHg as reported in mice of similar genetic background with normal ocular phenotypes (Savinova et al., 2001).

In comparing RGC densities across flat-mount naïve Cyp1b1^–/–^ and wildtype retina, no statistical difference was observed in retinal Brn3a cell density (Figure 1B, upper panel) between the two genotypes (Figure 1B; *F*_1_,_22_ = 0.23, *p* > 0.1). Using the optic nerves to compare node of Ranvier and paranode lengths (Figure 1C, upper image) across naïve wildtype and Cyp1b1^–/–^ subjects yielded no significant differences in node (Figure 1C, lower left; *F*_1_,_388_ = 0.36, *p* > 0.1) or paranode length (Figure 1C, lower right; *F*_1_,_388_ = 0.46, *p* > 0.1). Cyp1b1^–/–^ node and paranode lengths adhered to expected ranges for mice of equivalent age (Stahon et al., 2016). Although not quantified, sodium channels (Figure 1C, upper image; red) appeared present and normally distributed within the nodes. Lastly, to determine anterograde transport integrity and distal RGC axon/synaptic connectivity to/in the superior colliculus within Cyp1b1^–/–^ subjects; CTB, Vglut2, and Errβ label were independently compared across subjects. No differences were observed in the percent area fraction of CTB, Vglut2, and Errβ label in the superior colliculus of naïve Cyp1b1^–/–^ subjects compared to wildtypes (Figure 1D; CTB; *F*_1_,_22_ = 1.23, *p* > 0.1), Vglut2; *F*_1_,_22_ = 1.34, *p* > 0.1), and ERRβ, *F*_1_,_22_ = 1.23, *p* > 0.1).

### CYP1B1^–/–^ Accelerates Axonopathy in the Rretinal Projection Following Ocular Pressure Elevation

**FIGURE 2:**
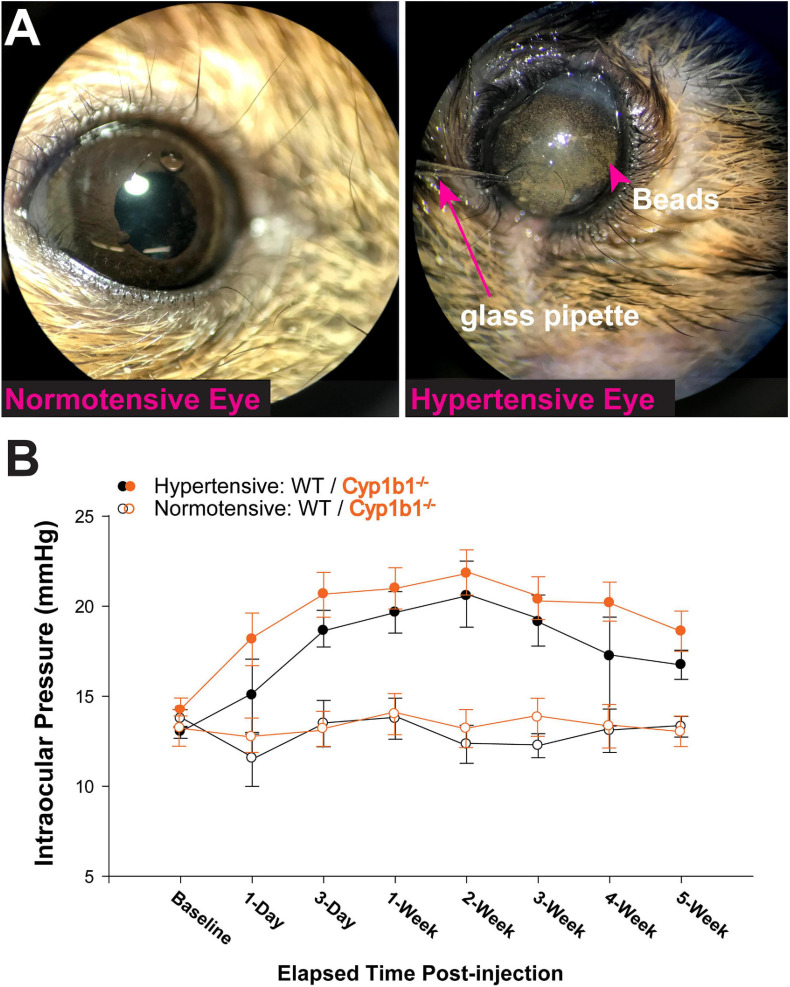
Microbead occlusion model. **(A)** Example image of procedure being performed. Left shows saline injected eye, right shows glass pipette insertion into anterior chamber of subject delivering a solution containing 8 μm magnetic microbeads. **(B)** Intraocular pressure (IOP) readings from Cyp1b1^–/–^ subjects (orange lines) and wildtype (black lines) following injection of microbeads (hypertensive and solid circles) or saline (normotensive and open circles) over 5 weeks (*n* = 24).

A single unilateral injection of magnetic microbeads (Figure 2A) significantly elevated IOP in both Cyp1b1^–/–^ and wildtype eyes by 38% for 5-weeks compared to the corresponding saline injected (normotensive) eyes (Figure 2B, *F*_3_,_26_ = 1.70, *p* > 0.1). Cyp1b1^–/–^ IOPs in the injected eye (hypertensive) (Figure 2B, solid circle- orange) was not significantly different from hypertensive wildtype eyes (Figure 2B, solid circle-black; *F*_1_,_26_ = 1.70, *p* > 0.1).

**FIGURE 3:**
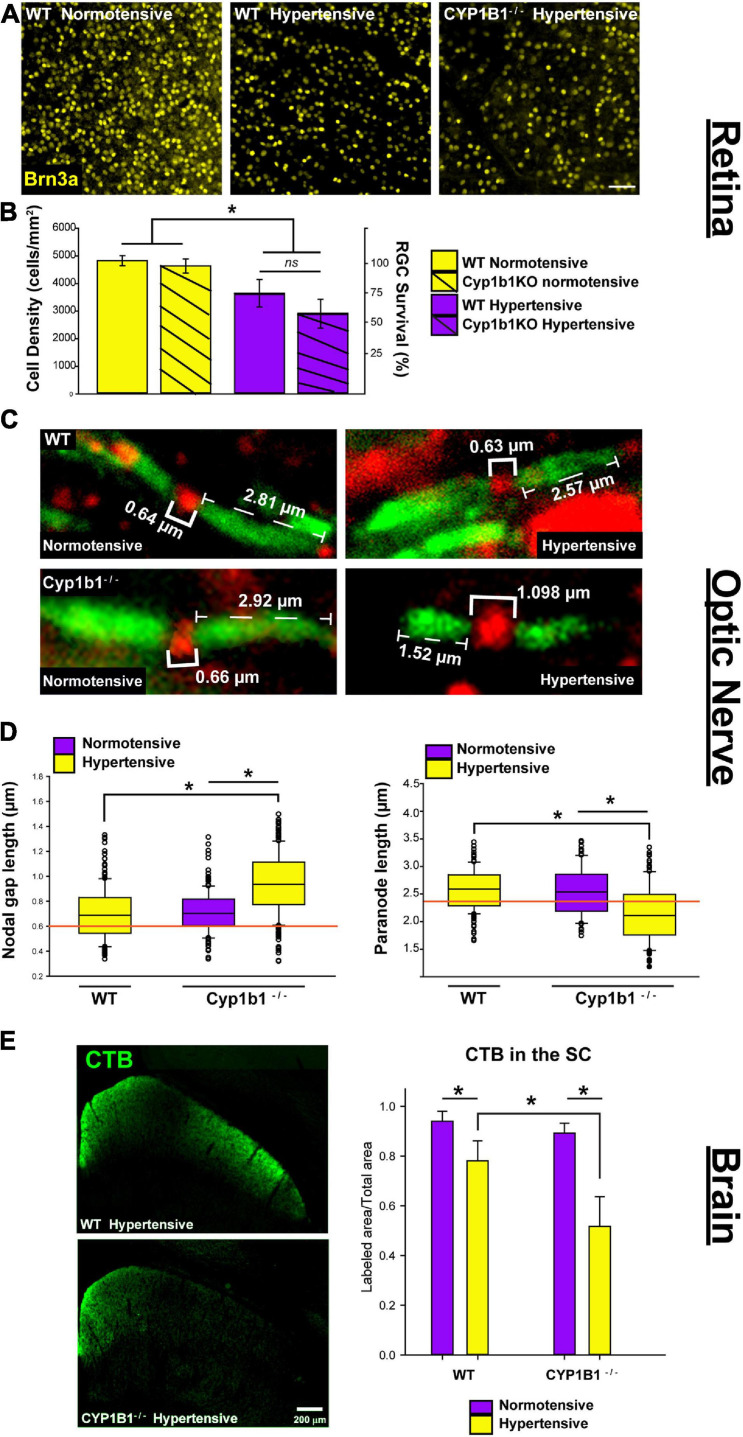
Cyp1b1^–/–^ accelerates axonopathy in the retinal projections following ocular pressure elevation. **(A)** Brn3a immunofluorescence in normotensive and hypertensive flat mount retina from wildtype and Cyp1b1^–/–^ subjects (scale bar = 50 μm). **(B)** Hypertensive Cyp1b1^–/–^ (solid magenta) and wildtype (dashed magenta) retina demonstrate a significant reduction in Brn3a density compared to normotensive wildtype (solid yellow) and Cyp1b1^–/–^ (dashed yellow) retina. Hypertensive Cyp1b1^–/–^ demonstrate no greater loss in Brn3a density compared to hypertensive wildtype retina. **(C)** Longitudinal optic nerve sections immunolabeled for Nav1.6 (red) and Caspr (green) to visualize nodes of Ranvier and adjacent paranode in normotensive and hypertensive Cyp1b1^–/–^ and wildtype optic nerves. Brackets indicate node and paranode length in microns. **(D)** Node lengths increased (left) while paranode lengths decreased (right) in hypertensive (yellow) Cyp1b1^–/–^ optic nerves following 5-week elevation in IOP compared to normotensive (magenta) nerves corresponding to the saline injected eye in the same animal. Nodes and paranode lengths associated with the hypertensive wildtype optic nerves were comparable to wildtype naïve (orange line; indicates average) and normotensive Cyp1b1^–/–^ (magenta) lengths. **(E)** Epifluorescent images contrasting CTB label in unilateral coronal sections through the superior colliculus of hypertensive Cyp1b1^–/–^ and wildtype subjects. Superior colliculi corresponding to hypertensive eyes (yellow) in both Cyp1b1^–/–^ and wildtype subjects demonstrate significant deficits in axonal transport evidenced by reduced CTB labels compared to colliculi corresponding to the saline injected normotensive eye (magenta). Collicular CTB drop-out was more pronounced in Cyp1b1^–/–^ subjects compared to wildtypes following equivalent period of IOP elevation. Asterisk denotes statistical significance (*p* < 0.01).

As summarized in Figure 3, we observed significant differences in the structural integrity of RGCs at the level of the retina, optic nerve, and superior colliculus following ocular hypertension in Cyp1b1^–/–^ and wildtype subjects. In the retina, following ocular hypertension, hypertensive Cyp1b1^–/–^ and hypertensive wildtype subjects exhibited substantial reductions (75 and 60%, respectively) in Brn3a density compared to saline injected controls (Figures 3A,B; *F*_3_,_44_ = 101.1, *p* < 0.01). However, hypertensive Cyp1b1^–/–^ retina did not exhibit any difference in Brn3a destiny compared to hypertensive wildtype retina (Figures 3A,B; *F*_3_,_44_ = 3.08, *p* = 0.09).

In the optic nerve, significant alterations in node and paranode lengths were observed (Figure 3C). Hypertensive Cyp1b1^–/–^ optic nerve nodes of Ranvier were 27% longer compared to nodes within the hypertensive wildtype optic nerve (Figure 3D left; *F*_1_,_446_ = 226.76, *p* < 0.05) and the contralateral normotensive Cyp1b1^–/–^ optic nerve (*F*_1_,_446_ = 203.23, *p* < 0.05). Hypertensive Cyp1b1^–/–^ paranode lengths were 21% shorter in comparison to hypertensive wildtype (Figure 3D right; *F*_1_,_446_ = 207.94, *p* < 0.05) and normotensive Cyp1b1^–/–^ optic nerves (*F*_1_,_446_ = 220.65, *p* < 0.05). Although not directly quantified, qualitatively, sodium channel density across axons and distribution within the node appeared consistent across groups and conditions.

Lastly, we compared the progression of anterograde transport deficits in the superior colliculus of Cyp1b1^–/–^ and wildtype mice following induced ocular hypertension (Figure 3E). Following the 5-weeks period of IOP elevation, the superior colliculus of both Cyp1b1^–/–^ and wildtype subjects demonstrated 45% (*F*_1_,_22_ = 299.57, *p* < 0.01) and 20% (*F*_1_,_22_ = 104.54, *p* < 0.01) depletion of CTB coverage, respectively. However, most interestingly, Cyp1b1^–/–^ demonstrated a 33% greater depletion in CTB transport in the superior colliculus compared to hypertensive wildtype subjects (*F*_1_,_22_ = 106.47, *p* < 0.01). Colliculi corresponding to the saline injected normotensive eyes in both subjects revealed >95% CTB coverage with statistical difference reflected across genotypes.

## Discussion

Axonopathy is one of the earliest hallmarks of neurodegeneration in glaucoma which encompasses degradation of active transport from the retina to the colliculus, and alterations in node of Ranvier morphology, both of which occur before outright axon degeneration in the optic nerve and soma loss in the retina (Dengler-Crish et al., 2014; Smith et al., 2016; Smith et al., 2018). In the current study, we showed evidence that absence of Cyp1b1 alone is insufficient to drive glaucomatous-like axonopathy and RGC neurodegeneration in the mouse visual system. This finding was not surprising given that these animals do not develop abnormal elevations in IOP over their lifespan. This seems to align with previous studies that also did not report abnormal IOPs in these mice, and provide evidence suggesting that the abnormal development in anterior eye structures is restricted to only modest focal points across these drainage structures and therefore are not substantial enough to alter the aqueous fluid drainage (Libby et al., 2003).

Our most interesting finding is that after a 5-weeks period of an equivalent pathological elevation in IOP, deficits in anterograde transport to the colliculus were more severe in Cyp1b1^–/–^ mice than in age-matched wildtype mice. Additionally, IOP elevation altered node of Ranvier morphology, where node gap lengths were longer and paranode lengths were shorter in Cyp1b1^–/–^ hypertensive optic nerves compared to equivalently stressed wildtype subjects. This observation is further intriguing given that alterations in node of Ranvier morphology are not typically reported following IOP elevation induced by microbead occlusion, but rather in naturally occurring animal models such as the DBA2/J mouse (Smith et al., 2018). It is important to note that despite the nuanced alterations in node/paranode morphology and the exacerbated transport loss, Cyp1b1^–/–^ subjects did not demonstrate any greater difference in RGC somal loss in the retina compared to wildtypes following ocular hypertension. Given that RGC degeneration in glaucoma occurs in a retrograde fashion occurring first in the distal axon and dying back toward cell bodies at varied rates, timing is critical. Our data represent a single time point following elevation whereby the percent change in RGC densities would have to be analyzed across multiple earlier and later time points to elucidate effects on RGC apoptosis in the retina. In any case, clinical manifestation and treatment strategies are not wholly left to preventing RGC loss and these exacerbated deficits in axonal transport and node of Ranvier alterations stand to have immense impact on RGC function.

In terms of possible mechanisms, our findings lack the support of additional literature therefore, much is left to speculation until further studies are conducted. Nevertheless, we assert that interesting linkages can be made across our findings by focusing on the bioactive metabolites produced by Cyp1b1. As mentioned, Cyp1b1 participates in the conversion of retinol to retinoic acid (RA) and metabolism of unbound arachidonic acid (AA) (Choudhary et al., 2004). Several studies have proposed various beneficial effects of RA in central nervous system injury noting RAs ability to downregulate pro-inflammatory cytokines such as IL-1β and TNFα in order to alter macrophage and microglial activation and reactive oxygen species production (Choi et al., 2005; Dheen et al., 2005; Xu and Drew, 2006; De La Rosa-Reyes et al., 2019) and in mediating RGC survival following optic nerve injury (Duprey-Díaz et al., 2016). RA has been shown to be released from NG2 cells in the central nervous system (Mey et al., 2005). NG2 cells play an intimate supporting role at the node of Ranvier where their processes are shown to insert (Serwanski et al., 2017) in order to serve as sensors to communicate changes in neuron environment in times of stress/injury (Wu et al., 2008). While it is not understood, we can speculate that RA release from NG2 cells at the node may provide additional support to mediate anti-inflammatory processes following injury or stress (Palenski et al., 2013). In the absence of Cyp1b1, diminished RA levels may lead to increased neuroinflammation (Falero-Perez et al., 2019) at the node altering their morphology in response to stress placed on axons following IOP elevation.

In addition, arachidonic acid (AA), a fatty acid that is released from cell membrane phospholipids following mechanical stimulation or hypoxia is partly metabolized by Cyp1B1. AA concentrations have been described in the retina, optic nerve, and brain and may play a role in other axonopathies/neurodegenerative disorders such as Alzheimer’s disease which share several similarities to glaucoma (Conquet et al., 1975; Corcoran et al., 2004). AA has been shown to influence synaptic functions by acting as a retrograde messenger stimulating opening Kv channels (Angelova and Müller, 2009). Given that Cyp1b1 appears localized within the inner plexiform layer of the retina, it would in theory allow for maintained high concentration of AA levels at the bipolar-RGC synapse. This may alter RGC activity in Cyp1b1^–/–^ projections whereby increased levels of AA could alter RGC excitability through retrograde synaptic signaling through inhibited sodium/potassium currents and synaptic transmission (Fraser et al., 1993). Additionally, AA has been described to be involved in tau hyperphosphorylation (King et al., 2000). AA activates several kinases, including protein kinase α, which directly contribute to increased tau phosphorylation levels (Kochs et al., 1993). Tau hyperphosphorylation is evident in the glaucomatous optic nerve and can disrupt axonal transport mechanisms (Wilson et al., 2016). It is possible that absence of Cyp1b1 exacerbates axonal transport deficits through increasing intra-axonal RGC tau hyperphosphorylation.

## Conclusion

Absence of Cyp1b1 alone is not sufficient to alter visual function but does increase RGC susceptibility to axonopathy following pressure elevation. Thus, Cyp1b1 may contribute to the ability of RGCs to respond to stress or injury through internal or external signaling mechanisms mediated through bioactive metabolites.

## Data Availability Statement

The original contributions presented in the study are included in the article/supplementary material, further inquiries can be directed to the corresponding author/s.

## Ethics Statement

The animal study was reviewed and approved by the Northeast Ohio Medical University Institutional Animal Care and Use Committee.

## Author Contributions

NA contributed extensively to experimental design, the data collection, reporting of initial results, and writing of the manuscript. BF and SD assisted with immunofluorescence assays and the data analysis. RB assisted in the data collection, interpretation of the results, and manuscript review. MS designed the study, performed and assisted in the data collection and analysis, interpreted results, and prepared the manuscript. All authors contributed to the article and approved the submitted version.

## Conflict of Interest

The authors declare that the research was conducted in the absence of any commercial or financial relationships that could be construed as a potential conflict of interest.

## References

[B1] Akarsu A. N. Turacli M. E. Aktan S. G. Barsoum-Homsy M. Chevrette L. Sayli B. S. (1996). A second locus (GLC3B) for primary congenital glaucoma (Buphthalmos) maps to the 1p36 region. *Hum. Mol. Genet.* 5 1199–1203. 8842741 10.1093/hmg/5.8.1199

[B2] Angelova P. R. Müller W. S. (2009). Arachidonic acid potently inhibits both postsynaptic-type Kv4.2 and presynaptic-type Kv1.4 I_A_ potassium channels. *Eur. J. Neurosci.* 29 1943–1950. 10.1111/j.1460-9568.2009.06737.x 19453640

[B3] Aponte E. P. Diehl N. Mohney B. G. (2010). Incidence and clinical characteristics of childhood glaucoma: a population-based study. *Arch. Ophthalmol*. 128 478–482. 10.1001/archophthalmol.2010.41.Incidence 20385945 PMC2885872

[B4] Baltan S. Inman D. M. Danilov C. A. Morrison R. S. Calkins D. J. Horner P. J. (2010). Metabolic vulnerability disposes retinal ganglion cell axons to dysfunction in a model of glaucomatous degeneration. *J. Neurosci.* 30 5644–5652. 10.1523/JNEUROSCI.5956-09.2010 20410117 PMC2884009

[B5] Bansal S. Leu A. N. Gonzalez F. J. Guengerich F. P. Chowdhury A. R. Anandatheerthavarada H. K. (2014). Mitochondrial targeting of cytochrome P450 (CYP) 1B1 and its role in polycyclic aromatic hydrocarbon-induced mitochondrial dysfunction. *J. Biol. Chem.* 289 9936–9951. 10.1074/jbc.M113.525659 24497629 PMC3975038

[B6] Bejjani B. A. Xu L. Armstrong D. Lupski J. R. Reneker L. W. (2002). Expression patterns of cytochrome P4501B1 (Cyp1b1) in FVB/N mouse eyes. *Exp. Eye Res.* 75 249–257. 12384088

[B7] Buters J. T. Sakai S. Richter T. Pineau T. Alexander D. L. Savas U. (1999). Cytochrome P450 CYP1B1 determines susceptibility to 7, 12-dimethylbenz[*a*]anthracene-induced lymphomas. *Proc. Natl. Acad. Sci. U. S. A.* 96 1977–1982. 10.1073/pnas.96.5.1977 10051580 PMC26722

[B8] Choi W.-H. Ji K.-A. Jeon S.-B. Yang M.-S. Kim H. Min K.-J. (2005). Anti-inflammatory roles of retinoic acid in rat brain astrocytes: suppression of interferon-γ-induced JAK/STAT phosphorylation. *Biochem. Biophys. Res. Commun*. 329 125–131. 15721283 10.1016/j.bbrc.2005.01.110

[B9] Choudhary D. Jansson I. Stoilov I. Sarfarazi M. Schenkman J. B. (2004). Metabolism of retinoids and arachidonic acid by human and mouse cytochrome P450 1b1. *Drug Metabol. Disposit.* 32 840–847. 10.1124/dmd.32.8.840 15258110

[B10] Conquet P. Plazonnet B. Le Douarec J. C. (1975). Arachidonic acid-induced elevation of intraocular pressure and anti-inflammatory agents. *Invest. Ophthalmol.* 14 772–775. 1184310

[B11] Corcoran J. P. T. So P. L. Maden M. (2004). Disruption of the retinoid signalling pathway causes a deposition of amyloid beta in the adult rat brain. *Eur. J. Neurosci.* 20 896–902. 15305858 10.1111/j.1460-9568.2004.03563.x

[B12] Crish S. D. Sappington R. M. Inman D. M. Horner P. J. Calkins D. J. (2010). Distal axonopathy with structural persistence in glaucomatous neurodegeneration. *Proc. Natl. Acad. Sci. U. S. A.* 107 5196–5201. 10.1073/pnas.0913141107 20194762 PMC2841892

[B13] Davis B. M. Crawley L. Pahlitzsch M. Javaid F. Cordeiro M. F. (2016). Glaucoma: the retina and beyond. *Acta Neuropathol.* 132 807–826. 10.1007/s00401-016-1609-2 27544758 PMC5106492

[B14] De La Rosa-Reyes V. Duprey M. V. Blagburn J. M. Blanco R. E. (2019). Retinoic acid application affects optic nerve microglia and macrophages after optic nerve injury in frog Rana pipiens. *FASEB J.* 33 450.6–450.6. 10.1096/fasebj.2019.33.1_supplement.450.6 ^∗∗∗^450.6-450.6, 33414642

[B15] Dengler-Crish C. M. Smith M. A. Inman D. M. Wilson G. N. Young J. W. Crish S. D. (2014). Anterograde transport blockade precedes deficits in retrograde transport in the visual projection of the DBA/2J mouse model of glaucoma. *Front. Neurosci.* 8:290. 10.3389/fnins.2014.00290 25278826 PMC4166356

[B16] Dheen S. T. Jun Y. Yan Z. Tay S. S. W. Ling E. A. (2005). Retinoic acid inhibits expression of TNF-α and iNOS in activated rat microglia. *GLIA* 50 21–31. 15602748 10.1002/glia.20153

[B17] Doshi M. Marcus C. Bejjani B. A. Edward D. P. (2006). Immunolocalization of CYP1B1 in normal, human, fetal and adult eyes. *Exp. Eye Res.* 82 24–32. 10.1016/j.exer.2005.04.016 15979611

[B18] Duprey-Díaz M. V. Blagburn J. M. Blanco R. E. (2016). Exogenous modulation of retinoic acid signaling affects adult RGC survival in the frog visual system after optic nerve injury. *PLoS One* 11:e0162626. 10.1371/journal.pone.0162626 27611191 PMC5017682

[B19] Falero-Perez J. Sorenson C. M. Sheibani N. (2019). Cyp1b1-deficient retinal astrocytes are more proliferative and migratory and are protected from oxidative stress and inflammation. *Am. J. Physiol. Cell Physiol.* 316 C767–C781. 10.1152/ajpcell.00021.2019 30892936 PMC6620579

[B20] Fraser D. D. Hoehn K. Weiss S. MacVicar B. A. (1993). Arachidonic acid inhibits sodium currents and synaptic transmission in cultured striatal neurons. *Neuron* 11 633–644. 10.1016/0896-6273(93)90075-3 8398152

[B21] Hoskins H. D. Shaffer R. N. Hetherington J. (1984). Anatomical classification of the developmental glaucomas. *Arch. Ophthalmol.* 102 1331–1336. 10.1001/archopht.1984.01040031081030 6477252

[B22] Kaur K. Mandal A. K. Chakrabarti S. (2011). Primary congenital glaucoma and the involvement of CYP1B1. *Middle East Afr. J. Ophthalmol.* 18 7–16. 10.4103/0974-9233.75878 21572728 PMC3085158

[B23] Khitri M. R. Mills M. D. Ying G.-S. Davidson S. L. Quinn G. E. (2012). Visual acuity outcomes in pediatric glaucomas. *J. Am. Assoc. Pediatr. Ophthalmol. Strabismus.* 16 376–381. 10.1016/j.jaapos.2012.05.007 22929453

[B24] King M. E. Gamblin T. C. Kuret J. Binder L. I. (2000). Differential assembly of human tau isoforms in the presence of arachidonic acid. *J. Neurochem.* 74 1749–1757. 10.1046/j.1471-4159.2000.0741749.x 10737634

[B25] Kochs G. Hummel R. Meyer D. Hug H. Marmé D. Sarre T. F. (1993). Activation and substrate specificity of the human protein kinase C α and ζ isoenzymes. *Eur. J. Biochem.* 216 597–606. 10.1111/j.1432-1033.1993.tb18179.x 8375396

[B26] Lambert W. S. Carlson B. J. Ghose P. Vest V. D. Yao V. Calkins D. J. (2019). Towards a microbead occlusion model of glaucoma for a non-human primate. *Sci. Rep.* 9:11572. 10.1038/s41598-019-48054-y 31399621 PMC6689098

[B27] Libby R. T. Smith R. S. Savinova O. V. Zabaleta A. Martin J. E. Gonzalez F. J. (2003). Modification of ocular defects in mouse developmental glaucoma models by tyrosinase. *Science* 299 1578–1581. 12624268 10.1126/science.1080095

[B28] Mandal A. K. Bhatia P. G. Bhaskar A. Nutheti R. (2004). Long-term surgical and visual outcomes in Indian children with developmental glaucoma operated on within 6 months of birth. *Ophthalmology* 111 283–290. 10.1016/j.ophtha.2003.05.027 15019376

[B29] Matt N. Ghyselinck N. B. Pellerin I. Dupé V. (2008). Impairing retinoic acid signalling in the neural crest cells is sufficient to alter entire eye morphogenesis. *Devel. Biol.* 320 140–148. 10.1016/j.ydbio.2008.04.039 18539269

[B30] Mey J. Morassutti D. J. Brook G. Liu R.-H. Zhang Y.-P. Koopmans G. (2005). Retinoic acid synthesis by a population of NG2-positive cells in the injured spinal cord. *Eur. J. Neurosci.* 21 1555–1568. 10.1111/j.1460-9568.2005.03928.x 15845083

[B31] Molotkov A. Molotkova N. Duester G. (2006). Retinoic acid guides eye morphogenetic movements via paracrine signaling but is unnecessary for retinal dorsoventral patterning. *Development* 133 1901–1910. 10.1242/dev.02328 16611695 PMC2833011

[B32] Murray G. I. Melvin W. T. Greenlee W. F. Burke M. D. (2001). Regulation, function, and tissue-specific expression of cytochrome P450 CYP1B1. *Annu. Rev. Pharmacol. Toxicol.* 41 297–316. 10.1146/annurev.pharmtox.41.1.297 11264459

[B33] Muskhelishvili L. Thompson P. A. Kusewitt D. F. Wang C. Kadlubar F. F. (2001). In situ hybridization and immunohistochemical analysis of cytochrome P450 1B1 expression in human normal tissues. *J. Histochem. Cytochem.* 49 229–236. 10.1177/002215540104900210 11156691

[B34] Palenski T. L. Sorenson C. M. Jefcoate C. R. Sheibani N. (2013). Lack of Cyp1b1 promotes the proliferative and migratory phenotype of perivascular supporting cells. *Lab. Invest.* 93 646–662. 10.1038/labinvest.2013.55 23568032 PMC3791926

[B35] Quigley H. A. Broman A. T. (2006). The number of people with glaucoma worldwide in 2010 and 2020. *Br. J. Ophthalmol.* 90 262–267. 16488940 10.1136/bjo.2005.081224 PMC1856963

[B36] Risner M. L. Pasini S. Cooper M. L. Lambert W. S. Calkins D. J. (2018). Enhanced ganglion cell excitability in glaucoma. *Proc. Natl. Acad. Sci.U.S.A.* 115 E2393–E2402. 10.1073/pnas.1714888115 29463759 PMC5877940

[B37] Sarfarazi M. Akarsu A. N. Hossain A. Turacli M. E. Aktan S. G. Barsoum-Homsy M. (1995). Assignment of a locus (GLC3A) for primary congenital glaucoma (Buphthalmos) to 2p21 and evidence for genetic heterogeneity. *Genomics* 30 171–177. 8586416 10.1006/geno.1995.9888

[B38] Savinova O. V. Sugiyama F. Martin J. E. Tomarev S. I. Paigen B. J. Smith R. S. (2001). Intraocular pressure in genetically distinct mice: an update and strain survey. *BMC Genet.* 2:12. 10.1186/1471-2156-2-12 11532192 PMC48141

[B39] Schlamp C. L. Li Y. Dietz J. A. Janssen K. T. Nickells R. W. (2006). Progressive ganglion cell loss and optic nerve degeneration in DBA/2J mice is variable and asymmetric. *BMC Neurosci.* 7:66. 10.1186/1471-2202-7-66 17018142 PMC1621073

[B40] Serwanski D. R. Jukkola P. Nishiyama A. (2017). Heterogeneity of astrocyte and NG2 cell insertion at the node of ranvier. *J. Comp. Neurol.* 525 535–552. 10.1002/cne.24083 27448245 PMC5177504

[B41] Simons E. S. Smith M. A. Dengler-Crish C. M. Crish S. D. (2021). Retinal ganglion cell loss and gliosis in the retinofugal projection following intravitreal exposure to amyloid-beta. *Neurobiol. Dis.* 147:105146. 10.1016/j.nbd.2020.105146 33122075

[B42] Smith M. A. Plyer E. S. Dengler-Crish C. M. Meier J. Crish S. D. (2018). Nodes of Ranvier in Glaucoma. *Neuroscience* 390 104–118. 10.1016/j.neuroscience.2018.08.016 30149050

[B43] Smith M. A. Xia C. Z. Dengler-Crish C. M. Fening K. M. Inman D. M. Schofield B. R. (2016). Persistence of intact retinal ganglion cell terminals after axonal transport loss in the DBA/2J mouse model of glaucoma. *J. Comp. Neurol.* 524 3503–3517. 10.1002/cne.24012 27072596 PMC5050057

[B44] Stahon K. E. Bastian C. Griffith S. Kidd G. J. Brunet S. Baltan S. (2016). Age-related changes in axonal and mitochondrial ultrastructure and function in white matter. *J. Neurosci.* 36 9990–10001. 10.1523/JNEUROSCI.1316-16.2016 27683897 PMC5039264

[B45] Stoilov I. Jansson I. Sarfarazi M. Schenkman J. B. (2001). Roles of cytochrome p450 in development. *Drug Metabol. Drug Interact.* 18 33–55. 11522124 10.1515/dmdi.2001.18.1.33

[B46] Su C. C. Liu Y. F. Li S. Y. Yanget J. J. Yen Y. C. (2012). Mutations in the CYP1B1 gene may contribute to juvenile-onset open-angle glaucoma. *Eye* 26 1369–1377. 10.1038/eye.2012.159 22878448 PMC3470042

[B47] Teixeira L. B. Zhao Y. Dubielzig R. R. Sorenson C. M. Sheibani N. (2015). Ultrastructural abnormalities of the trabecular meshwork extracellular matrix in Cyp1b1-deficient mice. *Vet. Pathol.* 52 397–403. 24879660 10.1177/0300985814535613 PMC4285769

[B48] Tham Y. C. Li X. Wong T. Y. Quigley H. A. Aung T. Cheng C. Y. (2014). Global prevalence of glaucoma and projections of glaucoma burden through 2040: a systematic review and meta-analysis. *Ophthalmology* 121 2081–2090. 10.1016/j.ophtha.2014.05.013 24974815

[B49] Vasiliou V. Gonzalez F. J. (2008). Role of CYP1B1 in glaucoma. *Annu. Rev. Pharmacol. Toxicol.* 48 333–358. 10.1146/annurev.pharmtox.48.061807.154729 17914928

[B50] Vincent A. L. Billingsley G. Buys Y. Levin A. V. Priston M. Trope G. (2002). Digenic inheritance of early-onset glaucoma: CYP1B1, a potential modifier gene. *Am. J. Hum. Genet.* 70 448–460. 10.1086/338709 11774072 PMC384919

[B51] Wilson G. N. Smith M. A. Inman D. M. Dengler-Crish C. M. Crish S. D. (2016). Early cytoskeletal protein modifications precede overt structural degeneration in the DBA/2J mouse model of glaucoma. *Front. Neurosci.* 10:494. 10.3389/fnins.2016.00494 27857681 PMC5093131

[B52] Wu Y. J. Tang Y. F. Xiao Z. C. Bao Z. M. He B. P. (2008). NG2 cells response to axonal alteration in the spinal cord white matter in mice with genetic disruption of neurofilament light subunit expression. *Mol. Neurodegener.* 3:18. 10.1186/1750-1326-3-18 18957081 PMC2584033

[B53] Xu J. Drew P. D. (2006). 9-cis-retinoic acid suppresses inflammatory responses of microglia and astrocytes. *J. Neuroimmunol*. 171 135–144. 16303184 10.1016/j.jneuroim.2005.10.004 PMC2825699

